# Saccade, Pupil, and Blink Responses in Rapid Eye Movement Sleep Behavior Disorder

**DOI:** 10.1002/mds.28585

**Published:** 2021-03-22

**Authors:** Julia E. Perkins, Annette Janzen, Felix P. Bernhard, Karén Wilhelm, Donald C. Brien, Jeff Huang, Brian C. Coe, David Vadasz, Geert Mayer, Douglas P. Munoz, Wolfgang H. Oertel

**Affiliations:** ^1^ Centre for Neuroscience Studies Queen's University Kingston Ontario Canada; ^2^ Department of Neurology Philipps‐University Marburg Germany; ^3^ Department of Biomedical and Molecular Sciences Queen's University Kingston Ontario Canada

**Keywords:** prodromal Parkinson's disease, eye movement, Parkinson's disease, biomarker

## Abstract

**Background:**

Parkinson's disease (PD) patients exhibit deficits in saccade performance, pupil function, and blink rate. Isolated REM (rapid eye movement) Sleep Behavior Disorder (RBD) is a harbinger to PD making them candidates to investigate for early oculomotor abnormalities as PD biomarkers.

**Objectives:**

We tested whether saccade, pupillary, and blink responses in RBD were similar to PD.

**Methods:**

RBD (n = 22), PD (n = 22) patients, and healthy controls (CTRL) (n = 74) were studied with video‐based eye‐tracking.

**Results:**

RBD patients did not have significantly different saccadic behavior compared to CTRL, but PD patients differed from CTRL and RBD. Both patient groups had significantly lower blink rates, dampened pupil constriction, and dilation responses compared to CTRL.

**Conclusion:**

RBD and PD patients had altered pupil and blink behavior compared to CTRL. Because RBD saccade parameters were comparable to CTRL, pupil and blink brain areas may be impacted before saccadic control areas, making them potential prodromal PD biomarkers. © 2021 The Authors. *Movement Disorders* published by Wiley Periodicals LLC on behalf of International Parkinson and Movement Disorder Society

A major challenge in Parkinson's disease (PD) research is the discovery of a disease‐modifying therapy.^1^, ^2^ This therapy would be most effective during prodromal PD. To successfully prove such a therapy effective, biomarkers for prodromal PD must be identified. Such biomarkers should: (1) be related to the disease process and easily measurable; (2) reflect progression of prodromal PD; and (3) be responsive to therapy. Here, we investigate whether saccade, pupil, and blink behavior can provide suitable biomarkers. To test this hypothesis, we chose patients with the parasomnia “isolated REM (rapid eye movement) Sleep Behavior Disorder” (RBD).^3^ RBD is characterized by the loss of muscle atonia during REM sleep accompanied by dream enactment. The annual rate of phenoconversion of RBD to PD or another alpha‐synucleinopathy disorder (αSYND) is approximately 6% and nearly 80% of RBD individuals will develop an αSYND within 10–15 years.^4^, ^5^, ^6^ For these reasons, RBD is considered a specific prodromal phenotype for PD^7^, ^8^, ^9^ and suitable for biomarker research.

PD is clinically diagnosed using cardinal motor symptoms, namely bradykinesia, muscle rigidity, and resting tremor.^10^ These symptoms are caused, at least in part, by the loss of dopaminergic neurons in the substantia nigra pars compacta (SNc).^11^, ^12^, ^13^, ^14^ An alternative is to objectively investigate oculomotor system abnormalities. Video‐based eye‐tracking provides a simple, non‐invasive, and effective way to assess brain function related to oculomotion, pupillary function, and blink rate. Measuring saccade, pupil, and blink behavior during sensory, motor, and cognitive tasks allows for the assessment of multiple brain circuits.^15^, ^16^, ^17^, ^18^ The interleaved pro‐ and anti‐saccade task (IPAST) pseudo‐randomly combines pro‐saccade (look towards a peripheral stimulus) and anti‐saccade (look in the opposite direction of peripheral stimulus) trials, which scrutinize areas that are implicated in PD, such as the basal ganglia (BG).^14^, ^15^, ^19^, ^20^, ^21^, ^22^ This task is also associated with pupil size changes related to locus coeruleus (LC) function.^23^ PD patients have specific deficits: increase in direction errors and slowed saccadic reaction time (SRT) during anti‐saccade trials, hypometric amplitudes during the pro‐saccade trials,^24^, ^25^ and dampened pupil constriction and dilation.^22^ The combination of saccade, pupil, and blink behavior have not yet been systematically studied in prodromal PD.

A recent study comparing anti‐saccade behavior between RBD and healthy, age‐matched controls (CTRL)^26^ reported increased direction errors on horizontal anti‐saccade trials for RBD patients. Here, we explore whether RBD patients have comparable deficits in saccadic, pupil, and blink behavior during IPAST to PD patients (here, and previously reported)^22^, ^24^, ^25^, ^27^, ^28^, ^29^ and reported RBD patients.^26^ Differences between RBD and CTRL identify potential prodromal PD biomarkers in this cross‐sectional study.

## Methods

Here, we provide only a brief description of methodology that is expanded in Supplementary Materials S1.

### Participants

This study was reviewed and approved by the human research ethics board of Queen's University, Canada and the Faculty of Medicine at the Phillips‐University of Marburg, Germany. Participant demographic and clinical assessment details are provided in Table S1.

#### RBD Patients

Twenty‐two RBD patients were recruited and screened with the REM Sleep Behavior Disorder Screening Questionnaire (RBDSQ).^30^ The diagnosis RBD was confirmed using the criteria of the International Classification of Sleep Disorders,^31^ and video‐assisted polysomnography (PSG).

#### PD Patients

Twenty‐two PD patients diagnosed by UK Brain Bank criteria^32^ were recruited and clinically examined using the Movement Disorder Society Unified Parkinson's Disease Rating Scale Part III (MDS‐UPDRS III) and the Montreal Cognitive Assessment (MoCA).

#### Controls

Seventy‐four healthy controls were recruited in Kingston, Canada. All controls were age‐matched with RBD and PD patients of the study. CTRL also completed the MoCA.

### Recording Apparatus

Eye position, pupil size, and eye blinks were measured with a video‐based monocular eye‐tracker (see Supplementary Materials S1).

### Interleaved Pro‐ and Anti‐ Saccade Task

**FIG. 1:**
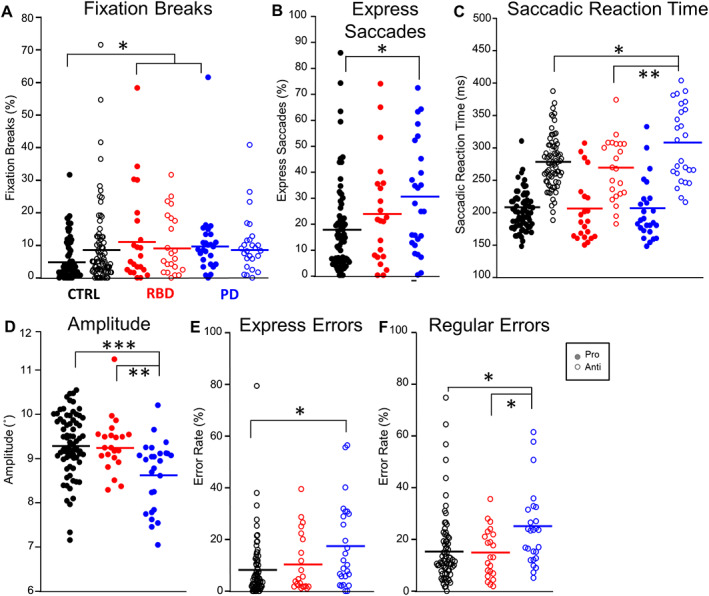
(**A**) Fixation breaks displayed by group during pro‐ (filled) and anti‐ (empty) saccade task. (**B**) Percent of express saccades during pro‐saccade trials (saccadic reaction time [SRT] >90 ms <140 ms). (**C**) Median SRTs during the pro (filled) and anti‐ (empty) saccade task. (**D**) Median amplitude of primary saccades initiated during correct pro‐saccade trials. (**E**) Percent of direction errors made during the express latency epoch (SRT >90 ms <140 ms) made across groups, and (**F**) percent of direction errors made during the regular latency epoch (SRT >140 ms) made across groups. One‐way ANOVA with a post hoc Tukey's honest significant difference (HSD) revealed significance between groups *P ≤ 0.05, **P ≤ 0.01, ***P ≤ 0.001. CTRL, healthy, age‐matched controls; RBD, rapid eye movement sleep behavior disorder; PD, Parkinson's disease. [Color figure can be viewed at wileyonlinelibrary.com]

Participants were seated in a dark room in front of a computer screen to perform IPAST (see Supplementary Materials S1, Fig. 1A). The pro‐saccade condition required an automatic visuomotor response (look at the stimulus), while the anti‐saccade condition required suppression of the automatic response and generation of a voluntary saccade in the opposite direction^15^, ^33^ (details in Supplementary Materials S1). A total of 240 trials were collected.

### Data Analysis

For each trial, eye movements, pupil size, and blink rate were categorized by an auto‐marking script (details in Supplementary Materials S1). All statistical comparisons were performed in SPSS using a one‐way, repeated measures ANOVA with a Tukey's honest significant difference (HSD) post hoc comparison unless stated otherwise.

## Results

### Saccade Metrics

#### Fixation Breaks

There was a significant difference in fixation breaks (Fig. 1A) between groups during pro‐saccade trials (F[2, 119] =5.483, *P* = 0.005). Post hoc comparison revealed that RBD and PD patients made significantly more fixation breaks than CTRL (RBD: *P* = 0.005; PD: *P* = 0.023). There were no significant differences during anti‐saccade trials (F[2, 119] =0.068, *P* = 0.934).

#### Saccadic Reaction Time

SRTs were broken down into two separate epochs: express (90 ms ≤ SRT ≤ 140 ms) and regular latency saccades (SRT > 140 ms) (see Supplementary Materials S1). There was a significant between group difference in express saccades in the pro‐saccade task (F[2, 119] =3.980, *P* = 0.021) (Fig. 1B). Post hoc comparison revealed that there was no significant difference between RBD patients and CTRL (*P* = 0.395), or RBD and PD (*P* = 0.537). PD patients made significantly more express saccades than CTRL (*P* = 0.019). Regular latency SRTs during pro‐saccade trials (Fig. 1C) did not differ significantly between groups (F[2, 119] =0.151 *P* = 0.860). Anti‐saccade RTs differed between groups (F[2, 119] =3.555, *P* = 0.032). Post hoc comparison revealed that PD patients had significantly longer SRTs than RBD (*P* = 0.013) and CTRL (*P* = 0.027).

#### Saccade Amplitude

Mean saccade amplitude differed significantly between groups (Fig. 1D) (F[2, 119] =9.047, *P* < 0.000). Post‐hoc comparison determined that PD patients had significantly smaller amplitude pro‐saccades compared to CTRL (*P* < 0.000) and RBD patients (*P* = 0.005).

#### Direction Errors

We separated direction errors into express (90 ms ≤ SRT ≤ 140 ms) (Fig. 1E) or regular (SRT > 140 ms) (Fig. 1F) latency. There was a significant difference between groups for express (F[2, 119] =4.322, *P* = 0.015) and regular (F[2, 119] =3.663, *P* = 0.029) latency direction errors. Post hoc comparison determined no difference in express latency errors made between RBD and CTRL (*P* = 0.813) or PD (*P* = 0.175). PD patients made more express errors than CTRL (*P* = 0.011). Post hoc comparison determined no difference between regular latency errors made between RBD and CTRL (*P* = 0.636). PD patients made more regular latency errors than RBD (*P* = 0.021) and CTRL (*P* = 0.040).

#### Pupil Metrics

**FIG. 2:**
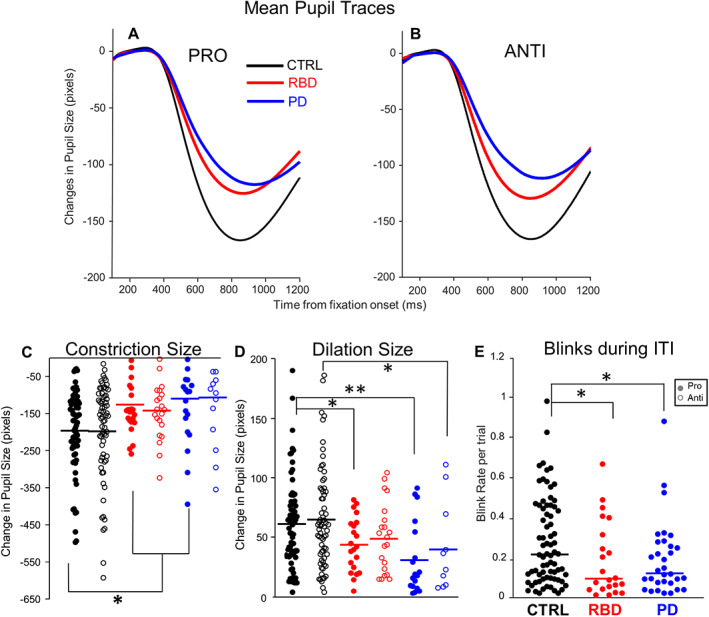
Mean pupil traces for each patient group are represented during the pro‐ (**A**) and anti‐ (**B**) saccade conditions over time. (**C**) Constriction size was defined as the pupil size at the greatest constriction after fixation appearance. (**D**) Dilation magnitude was defined as the pupil size at stimulus onset minus the pupil size at the time of greatest constriction during FI, reflecting the increase of pupil size after constriction. (**E**) Median blink rate during the inter‐trial interval (ITI). *P ≤ 0.05, **P ≤ 0.01, ***P ≤ 0.001. CTRL, healthy, age‐matched controls; RBD, rapid eye movement sleep behavior disorder; PD, Parkinson's disease. [Color figure can be viewed at wileyonlinelibrary.com]

Figure 2 shows averaged pupil responses during fixation and the gap period prior to target appearance. There were differences in amount of constriction and dilation between groups.

#### Constriction

There was a significant difference in pupil constriction size on pro‐saccade trials between groups (F[2, 104] =3.671, *P* = 0.029) (Fig. 2C). Post hoc comparison revealed that this was driven by a difference between RBD patients and CTRL (*P* = 0.038). RBD and PD patients did not differ from one another (*P* = 0.900). PD patients' pupils constricted significantly less than CTRL's during pro‐saccade trials (*P* = 0.035). There were group differences for constriction during anti‐saccade trials (F[2, 104] =2.834, *P* = 0.063).

#### Dilation

There was a significant difference between groups in pupil dilation during pro‐saccade trials (Fig. 2D) (F[2, 104] =6.684, *P* = 0.002). Post hoc comparison revealed a significant difference between RBD and CTRL patients (*P* = 0.045), and CTRL and PD patients (*P* = 0.001), but not RBD and PD (*P* = 0.216) during pro‐saccade trials. There was a significant difference between groups during the anti‐saccade trials (Fig. 2D) (F[2, 104] =4.346, *P* = 0.015). Post hoc comparison revealed there was no significant difference between RBD and CTRL (*P* = 0.234), and RBD and PD patients (*P* = 0.564) during the anti‐saccade trials. There was a significant difference between PD and CTRL (*P* = 0.018).

#### Blink Rate

There was a significant difference in blink rate between groups during the inter‐trial interval (ITI) (H [2]=6.957, *P* = 0.0155) (Fig. 2E). A post hoc pairwise comparison of groups determined that RBD (*P* = 0.0135) and PD (*P* = 0.024) patients made significantly fewer blinks than CTRL. There was no significant difference in blink rate between patients with RBD and PD (*P* = 0.336).

## Discussion

We demonstrated reduced pupil constriction and dilation and reduced blink rate for RBD as well as for PD in comparison to CTRL. Thus, pupil and blink behavior may be sensitive indicators for neurodegeneration in RBD. Likewise, changes in these two parameters may represent objective, quantifiable biomarkers for prodromal PD pathophysiology.

We replicated previous findings of pro‐ and anti‐saccadic behavior in PD.^24^, ^27^, ^28^, ^34^, ^35^ Specifically, PD patients had significantly longer SRTs during the anti‐saccade trials (Fig. 1C), made more express saccades on pro‐saccade trials (Fig. 1B), had significantly lower amplitude pro‐saccades (Fig. 1D), and made more express and regular latency direction errors on anti‐saccade trials (Fig. 1E,F, respectively). This is analogous to features presented in Lu et al,^35^ where both on‐ and off‐medication PD participants differed from CTRL aside from pro‐saccade latency, as shown here (Fig. 1C). The discrepancy between our findings and previously reported RBD patients^26^ in respect to direction errors in the anti‐saccade task needs further detailed and long‐term investigations (further discussion in Supplementary Materials S1). In line with previous PD studies,^22^, ^25^, ^28^ both RBD and PD patients demonstrated significantly less pupil constriction during pro‐saccade trials (Fig. 2C). However, only PD patients had significantly less dilation during anti‐saccade trials (Fig. 2D). PD patients made significantly fewer blinks compared to CTRL in previous studies.^10^, ^19^, ^36^, ^37^ Here, both patients groups’ blink rate was less than CTRL during the ITI (Fig. 2E). It is likely that natural or spontaneous blinks tended to occur during the ITI to not interfere with task performance. This mechanism may be altered in PD and RBD. The similarity in reduced blink rate in both RBD and PD patients suggests that this deficit may be independent of the dopaminergic system. In RBD, the decrease in the dopaminergic nigrostriatal system must, by definition, be below the threshold for manifestation of cardinal motor symptoms.

The impairment of RBD pupil and blink responses − comparable to PD patients − is consistent with the hypothesis that lower areas of the brain stem^38^ and LC^20^ are affected in RBD by underlying αSYND. Pupillary function is controlled by a circuitry between the LC and the parasympathetic and sympathetic pupil pathways,^22^ and the LC is also involved in blink behavior.^19^, ^39^ The clinical phenotype of isolated RBD (ie, no motor impairment) is considered to be associated with a lesion in the sublaterodorsal nucleus of the LC/subcoeruleus complex. Thus, it is likely that the noradrenergic neurons in the LC are affected by the underlying αSYND before the dopaminergic system. The next step is to determine when these ocular abnormalities arise within RBD patients, and if they progress alongside the disorder continuing towards phenoconversion.

## Author Roles

(1) Research project: A. Conception; B. Organization; C. Execution; (2) Data Collection; (3) Statistical Analysis: A. Design; B. Execution; C. Review and Critique; (4) Manuscript: A. Writing of the First Draft; B. Review and Critique.

J.E.P.: 1B, 1C, 2, 3A, 3B, 3C, 4A

A.J.: 1B, 1C, 2

F.P.B.: 2

K.W.: 2

D.C.B.: 3A

J.H.: 3A

B.C.C.: 3A

D.V.: 2

G.M.: 2

D.P.M.: 1A, 1B, 3C, 4B

W.H.O.: 1A, 1B, 3C, 4B

## Supporting information

**AppendixS1**: Supporting information
