# Supplementary material for: Saccade, Pupil, and Blink Responses in Rapid Eye Movement Sleep Behavior Disorder
Source: Mov Disord. 2021 Mar 22;36(7):1720–6. doi: 10.1002/mds.28585 (PMC8359943; doi:10.1002/mds.28585)
Supplement: Supplementary file 1 — AppendixS1: Supporting information [file MDS-36-1720-s001.docx]

# Supplementary Materials

# Methods

This study was reviewed and approved by the human research ethics board of Queen’s University, Canada and of the Faculty of Medicine at the Phillips-University of Marburg, Germany. All participants were given a letter of information detailing the study and provided written informed consent prior to participation. Eye-tracking was completed in two separate locations. CTRL were recruited through Hotel Dieu Hospital, Kingston, Canada, while RBD and PD patients were recruited through the Department of Neurology, University Clinic, Marburg, Germany. See Supplementary Table 1 for demographic and clinical information.

RBD Patients. 22 isolated RBD patients were recruited. They were first screened with the REM sleep behavior disorders Screening Questionnaire (RBD-SQ)^1^. The diagnosis RBD was confirmed using the criteria of the International classification of Sleep Disorders (American Academy of Sleep Medicine, 2005), and using video-assisted polysomnography (PSG). PSGs were rated by an experienced rater for the diagnosis of RBD^3^ at the Department of Neurology, University Clinic Marburg. RBD patients underwent clinical general and neurological examination including rating by Movement Disorder Society Unified Parkinson’s Disease Rating Score III (MDS-UPDRS III), Montreal Cognitive Assessment (MOCA) and an in-depth history of medication with focus on the use of antidepressants. Secondary-caused RBD (e.g.: due to medication) were not eligible to participate in the present study.

PD Patients. 22 PD patients diagnosed by the UK-Brain Bank criteria^4^ for PD were recruited and clinically examined using MDS-UPDRS III and MOCA just before completing IPAST. Four PD patients were classified as de novo (never received treatment) and thus investigated without previous therapy, 16 PD patients were in defined off-stage (no dopamimetic medication for at least 12 hours), and 8 patients were on standard dopamimetic medication (on-stage). Previous studies have revealed small, sometimes significant improvements in saccadic reaction time (SRT) and direction errors during anti-saccade trials in PD patients after taking dopaminergic medication.^5,6^ However, these effects are small and never strong enough to fully normalize function and so we did not insist that patients withdraw dopamimetic medication.

Controls. 74 healthy controls were recruited in Kingston, Ontario, Canada. All controls were age-matched with RBD and PD patients of the study. In order to keep statistical power, we chose not to sex-match CTRL to patients. These control participants were a part of a larger normative database where we were able to enroll a large amount of CTRL in order to gain an accurate representation of CTRL behavior, as with age, behavior becomes more variable. CTRL also completed the MOCA.

*Table 1.* Demographics and clinical score of participants

| Group | Number of Participants | Age at time of Measurement | MoCA Score | RBD Questionnaire Score | Hoehn and Yahr score | MDS-UPDRS III Score |
| --- | --- | --- | --- | --- | --- | --- |
| CTRL | 74 (53F) | Mean 66.51 ± 4.98 | Mean 27.71 ± 1.65 |  | - | - |
| RBD | 22 (4F) | Mean 67.08 ± 5.24 | Mean 27.94 ± 1.6 | Mean 9.61 ± 2.47 | 0 | Mean 2.16 ± 2.22 |
| PD | 22 (9F) | Mean 66.06 ± 5.17 | Mean 26.29 ± 3.91 |  | Mean 2.05 ± 0.58 | Mean 25.39 ±14.24 |

## Recording and Apparatus

Stimulus presentation and data acquisition were controlled by Eyelink Experiment Builder and Eyelink software. Eye position, pupil size, and blink rate were measured at a rate of 500Hz with a video-based monocular eye tracker (Eyelink-1000 Plus, SR Research Ltd, Osgoode, ON, Canada). Eye-trackers were identical at the two research facilities (Kingston and Marburg) and their specifications were monitored closely. Stimuli were presented on a 17-inch LCD monitor at a screen resolution of 1280×1024 pixels (60Hz refresh rate), subtending a viewing angle of 32×26°, and distance from the eyes to the monitor and infrared camera was set at 60cm, the optimal eye camera-eye distance. All recordings and calibrations were done monocularly based on the right eye. Eye position was first calibrated using a nine-point grid (eight around the periphery and one central). The stimuli were flashed randomly around the screen and the participant had to fixate on each until the next stimulus appeared. After calibration, the process was repeated to validate that the average error between fixation and stimulus was <1° and that no loss of eye tracking occurred. To ensure that significant differences reported were not due to location differences, both video-based eye-tracking apparatuses underwent regular and repeated rigorous testing to ensure consistency between machines. This testing involved a spectrometer to guarantee equal luminance emitted by the eye-trackers’ screens as to not effect pupil baseline, constriction, and dilation levels. All data were collected within a windowless testing room, within respective hospitals, with all lights turned off during data collection to ensure the only luminance produced was by the computer monitor.

## Interleaved Pro- and Anti- Saccade task

Participants were seated in a dark room in front of a computer screen and performed IPAST which is illustrated in supplementary Fig. 1A.


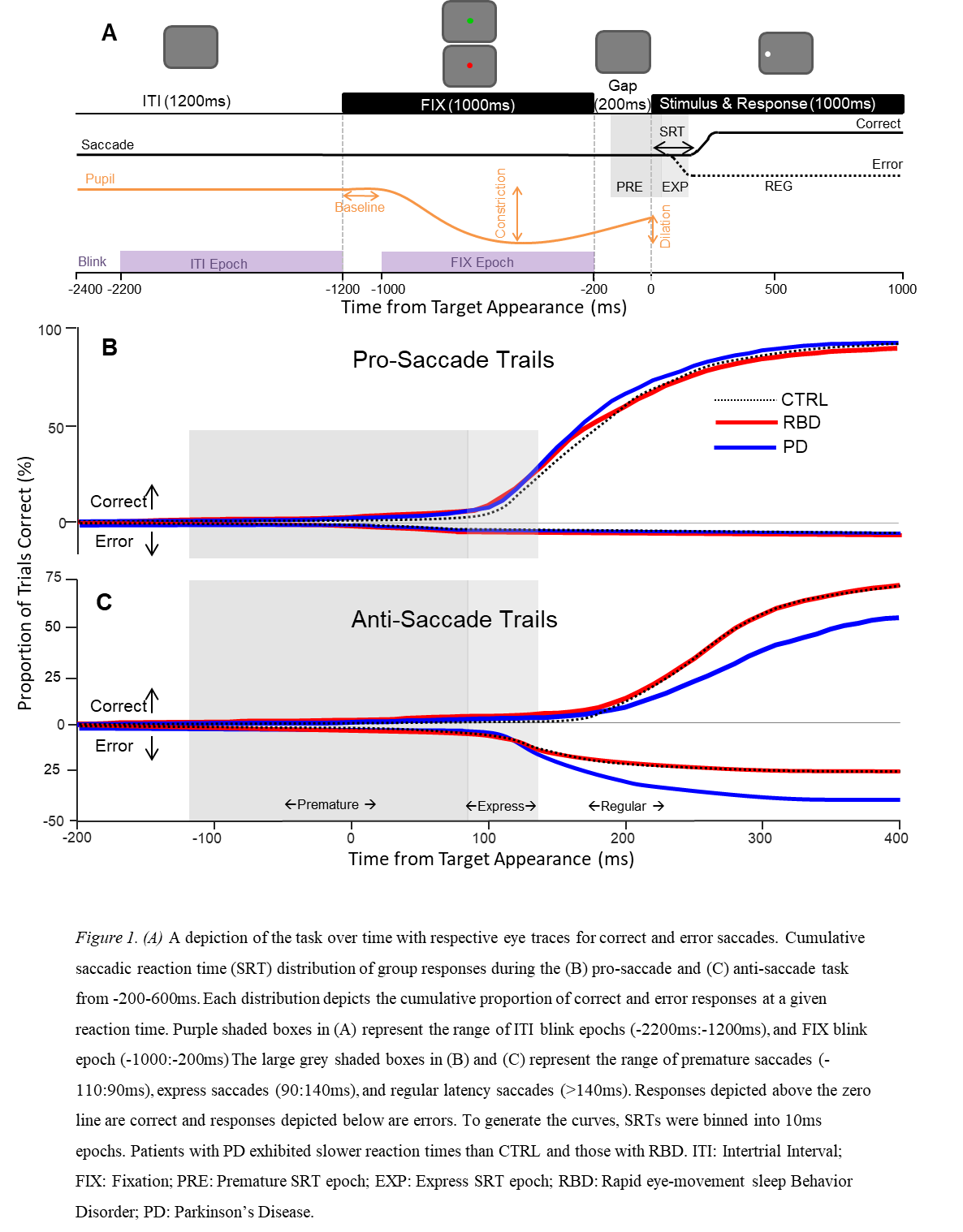


We chose this task as the pro-saccade condition requires an automatic visuomotor response, while the anti‐saccade condition requires suppression of the automatic saccade and generation of a voluntary response in the equal and opposite direction from the stimulus.^7,8^ PD patients have been known to exhibit deficits within this task.^9^ Each trial began with 1200ms of an Inter-trial Interval (ITI) consisting of a black background (0.1cd/m^2^) after which a central fixation point (FIX) (0.5° diameter, ~44 cd/m^2^) appeared. The task condition was revealed via FIX color (pro-saccade: green FIX; anti-saccade: red FIX; luminance level matched). The color-coded FIX lasted for 1000ms and then disappeared and was followed by a 200ms gap. Following the gap, a white peripheral stimulus appeared (0.5° diameter, ~62 cd/m^2^) to the left or to the right of the FIX (10˚ eccentricity on the horizontal axis) (Fig 1A). For pro-saccade trials (green FIX), participants were instructed to look towards the peripheral stimulus as soon as it appeared. For anti–saccade trials (red FIX), participants were instructed to look in the equal and opposite direction of the stimulus as soon as it appeared. Trial condition (pro- or anti-saccade) and stimulus location (left or right) were pseudo-randomly interleaved for each block of 120 trials. After every 40 trials, a drift-correct procedure was implemented to ensure the eye-tracking calibration remained accurate and the participant had not shifted. Participants’ fixation had to be <1˚from the drift-correct stimulus. If they exceeded 1˚, recalibration and validation were completed. The experiment was run twice, with a brief break in between runs, for a maximum of 240 trials per participant.

*Data Analysis*

Each trial and all eye movements were categorized by an auto-marking script written in MatLab (the MathWorks Inc., Natick, MA) and were manually verified to ensure accuracy and consistency. Trials could be excluded if eye tracking was lost (e.g., prolonged loss of eye tracking during trial greater than typical blink duration), if there was no saccade, if a saccade was made to a random location, if fixation upon FIX was never made, if fixation was broken, or other such reasons that rendered the trial non-viable.

Saccadic reaction time (SRT) refers to the time from peripheral stimulus appearance to the initiation of the first saccade. Both direction error rates (defined as saccades that followed the wrong instruction) and SRTs were calculated as per-participant medians, which were then compared across groups. Premature saccades occurred during the gap period (defined as saccades made within -110ms to +90ms of peripheral stimulus appearance the actual time of the gap period plus a 90ms delay in neural transmission of visual and motor signals)^8^ and had an equally likelihood to be correct or incorrect.^10,11^ Saccades away from FIX that made from -910ms to -110ms of target appearance were deemed as fixation breaks and categorized separately to compare between groups. Express saccades were defined as saccades made within 90ms-140ms of the stimulus appearing.^12,13^ The 90ms cut off was selected as it is consistent with the delay in neural transmission mentioned above.^8^ Any saccade initiated after 140ms was considered a regular latency saccade. Using these epochs, we then differentiated different error types: express latency direction errors and regular latency direction errors. Only those trials that met the following criteria were analyzed as correct responses: there was no direction error; SRT was >90ms; no blinks were made; no saccades >2° during the fixation period were made before stimulus appearance (i.e., fixation break); participants began fixating on FP within 150ms after it appeared; and pupil velocity during the fixation period did not reach unnatural values indicating excessively noisy recording. After all of these criteria were met, a participant’s data were included in the analysis if they produced at least 10 viable trials for each of the pro- and anti- saccade conditions.

Amplitude was defined as the linear distance the eye travels from the starting position of the initial saccade, to the end point of the initial saccade. Amplitude was only analyzed for these initial saccades, and only for pro-saccade trials. Pro-saccade trials were only analyzed as participants performed a saccade towards a visible stimulus. Where, during anti-saccades, participants performed saccades in the opposite direction of the stimulus, where there was no landmark for accuracy. This, in turn, makes anti-saccade latency much more variable, and unreliable for statistical purposes.

*Pupil Analysis*

To investigate changes in pupil diameter, we baseline-corrected pupil size.^14,15^ For each trial, original pupil diameter was subtracted from the baseline pupil diameter. We analyzed both constriction and dilation pupil responses (Supplementary Fig. 1A). Constriction size was defined as the pupil size at the greatest constriction after FIX appearance. Dilation magnitude was defined as pupil size at the time of peripheral stimulus appearance minus the pupil size at the time of greatest constriction during FIX, reflecting the increase of pupil size after constriction.

*Blink Analysis*

During video eye-tracking, accurate tracking data was lost when the eye lid closes. Understanding this loss of data is a means to measuring blink behavior. The timing and duration of the data loss was used to categorize the events as blinks or other data loss. Data loss that was shorter than 100ms or longer than 600ms were not indicative of a natural blink and were not used, and data loss with a stereotypical duration of 100 - 600ms were designated as eyeblinks. Blink frequency was investigated during 2 epochs (Fig. 1A): the intertrial interval (ITI: -2200 to -1200ms before target appearance); and the fixation epoch (FIX: -1000 to -200ms before target appearance). Blinks which overlapped the two epochs or took place outside these two epochs were not further analyzed here.

*Statistical Analysis*

All statistical comparisons were performed in SPSS using a one-way, repeated measures ANOVA with a Tukey’s HSD post hoc comparison unless stated otherwise. Due to the non-normal distribution of the blink rate data, determined by a Kolmogorov-Smirov test (p=0.001), we completed a Kruskal-Wallis test here.

**Supplementary Results**

*Saccade Distributions*

Supplementary Fig 1B and C illustrates the distributions of SRTs for correct saccades and direction errors in the pro- and anti-saccade conditions for each group: CTRL (black dotted), RBD (red), PD (blue). Curves above the horizontal axis (0-line) represent mean correct saccades from the time from target appearance, whereas curves below represent mean direction errors from the time of target appearance. Highlighted in increasing shades of grey are saccade latency epochs: Premature (-110ms to +90ms), Express (90ms to 140ms), and regular (140ms onwards). Though these figures are qualitative in nature, we were able to observe substantial differences in errors rates and reaction times between groups, particularly during anti-saccade trials. Thus, we further separated SRTs, direction errors, and other metrics for detailed analyses to determine significance.

**Supplementary Discussion**

We were able to replicate previous findings of pro- and anti-saccadic behavior in PD patients.^5,6,16,17^ Specifically, PD patients had significantly longer SRTs during the anti-saccade trials (Fig. 1C), made more express saccades (Fig. 1B), had significantly lower amplitude in pro-saccade trials (Fig. 1D), and made more direction errors during the anti-saccade trials in both the express and regular latency epochs (Fig. 1F & G, respectively). In line with previous PD studies,^6,9,15^ both RBD and PD patients demonstrated significantly less pupil constriction during the FIX epoch of pro-saccade trials (Fig. 2C). However, only PD patients had significantly less dilation during anti-saccade trials (Fig. 2D). We report, for the first time, that RBD patients show a similar dysfunction (dampening) in pupil measures as PD patients. PD patients make significantly less blinks compared to CTRL in previous studies.^18–21^ Here, both patients groups blink significantly less than CTRL during ITI (Fig. 2E). It is likely that natural or spontaneous blinks tended to occur during the ITI to not interfere with task performance. This mechanism may be altered in PD and RBD.

*Saccade Behavior*

Interestingly, for saccadic behavior, only the percentage of fixation breaks (Fig. 1A) differed between CTRL and patients. We did not replicate a direction error increase for RBD patients during anti-saccade trials as reported previously by Hanuška et al (2019).^22^ Compared to our study, their experimental paradigm did not contain an Inter-Trial-Interval. They investigated a higher number of RBD patients than in our study, they included only de novo PD patients, and fewer CTRL. Their RBD patients showed a wide range of MDS-UPDRS III scores and a slightly lower MOCA score than the RBD cohort reported here. Thus, it may be that the observed increase in direction errors indicates that their RBD patients^35^ were very close to phenoconversion, or – alternatively – the result was related to mildly impaired cognitive function, including a dysfunction of the DLPFC. The first hypothesis implies that direction errors in horizontal anti-saccades should be further investigated in long-term follow-up studies as a potential indicator for imminent phenoconversion of RBD patients. The study by Hanuška et al (2019) and our study are both cross-sectional and require follow-up investigations to test this hypothesis. Further studies may show whether RBD patients with increased direction errors during IPAST will develop another alpha-synucleinopathies rather than PD. A further open question is whether the changes in the ocular parameters observed in the RBD patient group depend on the degree of neurodegeneration in the nigrostriatal system as visualized by dopamine transport ligand binding.

## Limitations

While the results present potential prodromal PD biomarkers, there are limitations. First, some of the parameters did not replicate previous studies in PD patients, such as pupillary constriction during anti-saccade trials. This may be due to keeping some PD patients on their medication, instead of testing during a defined off-medication period. Performance on cognitive tasks follows an inverted U-shaped response with increasing dopamine, including that introduced into the system via medication (i.e. levodopa).^23^ Thus, too little and too much dopamine within the system is detrimental to performance both in patients and CTRL.^5,24^ Though, as previously reported,^6^ there is a small difference between on and off-states in saccadic behavior for PD patients,^6,25^ it is not clarified whether this extends to the pupillary and blink systems. Further studies should be conducted to test this effect in both systems.

Another limitation is that CTRL and the patient populations were recruited at two separate geographic sites. Although this meant different operators collected the data at the two sites, we ensured that both experimental set-ups were virtually identical, including the same model of eye-tracker, a hospital room without windows to ensure comparable global luminance, and near identical verbal instructions, except language used: English for the CTRL; German for patient groups.

Lastly, it would be preferable to test this hypothesis in another prodromal PD population to determine whether the effects seen here are due to a prodromal state of PD – pupillary and blink-rate systems being affected before more predominant motor disturbances occur.

**References**

1. Stiasny-Kolster, K. *et al.* The REM sleep behavior disorder screening questionnaire - A new diagnostic instrument. *Mov. Disord.* **22**, 2386–2393 (2007).

2. Westchester: American Academy of Sleep Medicine. International Classification of Sleep Disorders. *Diagnostic Coding Man.* (2005).

3. Mayer, G. *et al.* Quantification of tonic and phasic muscle activity in REM sleep behavior disorder. *J. Clin. Neurophysiol.* **25**, 48–55 (2008).

4. Gibb, G. & Lees, A. J. The relevance of the Lewy body to the pathogenesis of idiopathic Parkinson’s disease. *Neurosurgery, and Psychiatry* **51**, 745–752 (1988).

5. Hood, A. J. *et al.* Levodopa slows prosaccades and improves antisaccades: an eye movement study in Parkinson’s disease. *J Neurol Neurosurg Psychiatry* **78**, 565–570 (2007).

6. Cameron, I. G. M. M. *et al.* Impaired executive function signals in motor brain regions in Parkinson’s disease. *Neuroimage* **60**, 1156–1170 (2012).

7. Munoz, D. P. & Everling, S. Look away: the anti-saccade task and the voluntary control of eye movement. *Nat. Rev. Neurosci.* (2004). doi:10.1038/nrn1345

8. Coe, B. C. & Munoz, D. P. Mechanisms of saccade suppression revealed in the anti-saccade task. *Philos. Trans. R. Soc. Lond. B. Biol. Sci.* **372**, 20160192 (2017).

9. Chan, F., Armstrong, I. T., Pari, G., Riopelle, R. J. & Munoz, D. P. Deficits in saccadic eye-movement control in Parkinson’s disease. *Neuropsychologia* **43**, 784–796 (2005).

10. Marino, R. A. *et al.* Linking visual response properties in the superior colliculus to saccade behavior. *Eur. J. Neurosci.* **35**, 1738–1752 (2012).

11. Bell, A. H., Meredith, M. A., Van Opstal, A. J. & Munoz, D. P. Stimulus intensity modifies saccadic reaction time and visual response latency in the superior colliculus. *Exp. Brain Res.* **174**, 53–59 (2006).

12. Fischer, B. & Ramsperger, E. Human express saccades: effects of randomization and daily practice. *Exp. Brain Res.* **64**, 569–578 (1986).

13. Kingstone, A. & Klein, R. M. What are human express saccades? *Percept. Psychophys.* **54**, 260–73 (1993).

14. Wang, C.-A., Brien, D. C. & Munoz, D. P. Pupil size reveals preparatory processes in the generation of pro-saccades and anti-saccades. *Eur. J. Neurosci.* **41**, 1102–1110 (2015).

15. Wang, C. A., McInnis, H., Brien, D. C., Pari, G. & Munoz, D. P. Disruption of pupil size modulation correlates with voluntary motor preparation deficits in Parkinson’s disease. *Neuropsychologia* **80**, 176–184 (2016).

16. Cameron, I. G. M., Watanabe, M., Pari, G. & Munoz, D. P. Executive impairment in Parkinson’s disease: Response automaticity and task switching. *Neuropsychologia* **48**, 1948–1957 (2010).

17. Amador, S. C., Hood, A. J., Schiess, M. C., Izor, R. & Sereno, A. B. Dissociating cognitive deficits involved in voluntary eye movement dysfunctions in Parkinson’s disease patients. *Neuropsychologia* **44**, 1475–1482 (2006).

18. Deuschl, G. & Goddemeier, C. Spontaneous and reflex activity of facial muscles in dystonia, Parkinson’s disease, and in normal subjects. *J. Neurol. Neurosurg. Psychiatry* **64**, 320–324 (1998).

19. Jongkees, B. J. & Colzato, L. S. Spontaneous eye blink rate as predictor of dopamine-related cognitive function—A review. *Neurosci. Biobehav. Rev.* **71**, 58–82 (2016).

20. Jankovic, J. Parkinson’s disease: clinical features and diagnosis. *J. Neurol. Neurosurg. Psychiatry* **79**, 368–376 (2008).

21. Mavridis, M., Degryse, A.-D., Lategan, A. J., Marien, M. R. & Colpaert, F. C. Effects of locus coeruleus lesions on parkinsonian signs, striatal dopamine and substantia nigra cell loss after 1-methyl-4-phenyl-1,2,3,6-tetrahydropyridine in monkeys: A possible role for the locus coeruleus in the progression of Parkinson’s disease. *Neuroscience* **41**, 507–523 (1991).

22. Hanuška, J. *et al.* Eye movements in idiopathic rapid eye movement sleep behaviour disorder: High antisaccade error rate reflects prefrontal cortex dysfunction. *J. Sleep Res.* **28**, (2019).

23. Meder, D., Herz, D. M., Rowe, J. B., Lehéricy, S. & Siebner, H. R. The role of dopamine in the brain - lessons learned from Parkinson’s disease. *NeuroImage* **190**, 79–93 (2019).

24. Duka, T. & Lupp, A. The effects of incentive on antisaccades: Is a dopaminergic mechanism involved? *Behav. Pharmacol.* **8**, 373–382 (1997).

25. Lu, Z., Buchanan, T., Kennard, C., FitzGerald, J. J. & Antoniades, C. A. The effect of levodopa on saccades – Oxford Quantification in Parkinsonism study. *Park. Relat. Disord.* **68**, 49–56 (2019).
